# Carbon dioxide reduction by photosynthesis undetectable even during phytoplankton blooms in two lakes

**DOI:** 10.1038/s41598-023-40596-6

**Published:** 2023-08-19

**Authors:** Karla Münzner, Silke Langenheder, Gesa A. Weyhenmeyer, Bianka Csitári, Eva S. Lindström

**Affiliations:** Division of Limnology, Department of Ecology and Genetics, Uppsala University, Uppsala, Sweden

**Keywords:** Climate sciences, Limnology

## Abstract

Lakes located in the boreal region are generally supersaturated with carbon dioxide (CO_2_), which emerges from inflowing inorganic carbon from the surrounding watershed and from mineralization of allochthonous organic carbon. While these CO_2_ sources gained a lot of attention, processes that reduce the amount of CO_2_ have been less studied. We therefore examined the CO_2_ reduction capacity during times of phytoplankton blooms. We investigated partial pressure of CO_2_ (*p*CO_2_) in two lakes at times of blooms dominated by the cyanobacterium *Gloeotrichia echinulata* (Erken, Sweden) or by the nuisance alga *Gonyostomum semen* (Erssjön, Sweden) during two years. Our results showed that *p*CO_2_ and phytoplankton densities remained unrelated in the two lakes even during blooms. We suggest that physical factors, such as wind-induced water column mixing and import of inorganic carbon via inflowing waters suppressed the phytoplankton signal on *p*CO_2_. These results advance our understanding of carbon cycling in lakes and highlight the importance of detailed lake studies for more precise estimates of local, regional and global carbon budgets.

## Introduction

Lakes in the boreal region are generally supersaturated with carbon dioxide (CO_2_) and, thus, net sources of CO_2_ to the atmosphere^1,2^. We know that much of the supersaturation emerges from import of inorganic carbon from the surrounding catchment^3^, including groundwater inflow^4,5^, and from mineralization of imported organic carbon^6–9^. On the other hand, processes that reduce the amount of CO_2_ in the water, like photosynthesis, are much less studied in this context. However, recent studies suggest that CO_2_ uptake by algae can have a stronger influence on CO_2_ emissions from lakes, even boreal ones, than previously thought^10,11^. Therefore, determining the scale of algal CO_2_ fixation on lake CO_2_ concentrations is a crucial step towards understanding the carbon cycling in those lakes and predicting their contribution to climate change.

We aimed here to get a better understanding of the role of phytoplankton in lake carbon cycling in the boreal region by assessing relations between phytoplankton densities and the partial pressure of CO_2_ (*p*CO_2_) in two lakes in which two different types of phytoplankton blooms occurred, i.e. blooms of cyanobacteria and blooms of raphidophytes. Phytoplankton communities in boreal lakes are commonly dominated by cryptophytes, cyanobacteria, chrysophytes, diatoms and raphidophytes^12,13^. Cyanobacteria blooms mostly occur in eutrophic non-colored lakes^14^, while humic lakes are more susceptible to blooms of flagellates, especially from the nuisance alga *Gonyostomum semen*^15–17^. Both cyanobacteria and *G. semen* blooms are expected to increase as a result of higher temperatures and nutrient concentrations due to increased runoff in a changing climate^15,18–22^. That makes them perfect model organisms to assess the impact of algal blooms on lake carbon cycling.

To fulfill our aim, we used *p*CO_2_ monitoring data from two Swedish lakes within the Swedish Infrastructure for Ecosystem Science (SITES): a humic lake (Erssjön) in Västergötland (spring – autumn 2017 and 2018), and a mesotrophic non-humic lake (Erken) in Uppland (spring – autumn 2018). The former one is subject to blooms of the raphidophyte *G. semen,* and the latter to blooms of the cyanobacterium *Gloeotrichia echinulata* in summer and diatoms in spring and autumn. We identified phytoplankton blooms based on chlorophyll *a* measurements and *G. semen* and *G. echinulata* densities. This was done with the help of water chemistry and phytoplankton monitoring data, as well as quantitative PCR (qPCR) for *G. semen* specifically. We hypothesized that a) *p*CO_2_ in Erssjön decreases with increasing *G. semen* densities, and b) *p*CO2 in Erken decreases with increasing *G. echinulata* densities.

## Results

### Algal blooms and phytoplankton community composition

**Figure 1:**
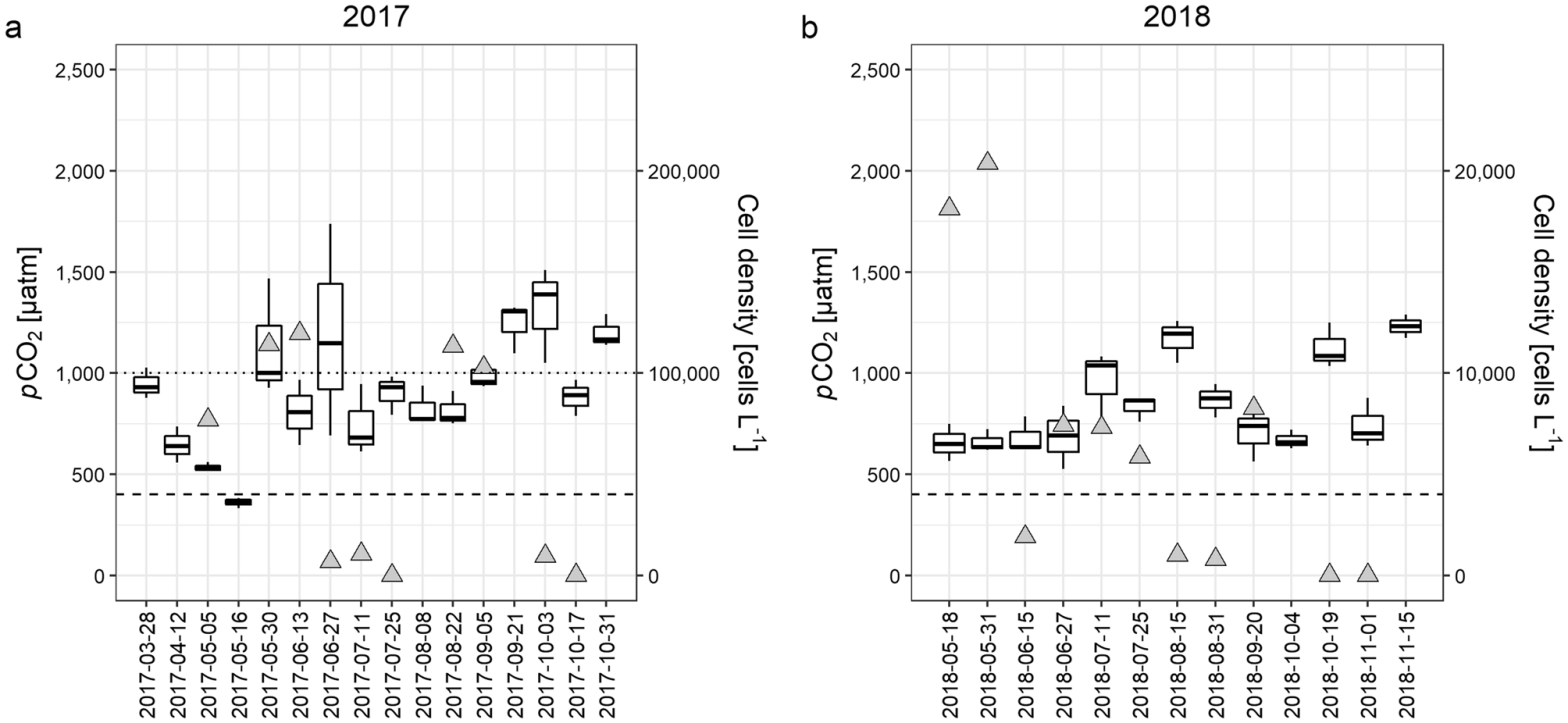
Lake *p*CO_2_ and *G. semen* densities based on qPCR results in Erssjön in 2017 (**a**) and 2018 (**b**). For each date, results from 3 sampling locations (*p*CO_2_ measurements, 4 replicates per sampling spot) or one sampling location (*G. semen* abundance) are shown. The boxes symbolize 50% of the data and the line is the median. Boxplots = *p*CO_2_, grey triangles = *G. semen* abundance, dashed line = *p*CO_2_ equilibrium (400 µatm), dotted line = cell number threshold indicating a phytoplankton bloom (100,000 cells L^−1^). Note the different scales in cell densities in (**a**) and (**b**).

**Figure 2:**
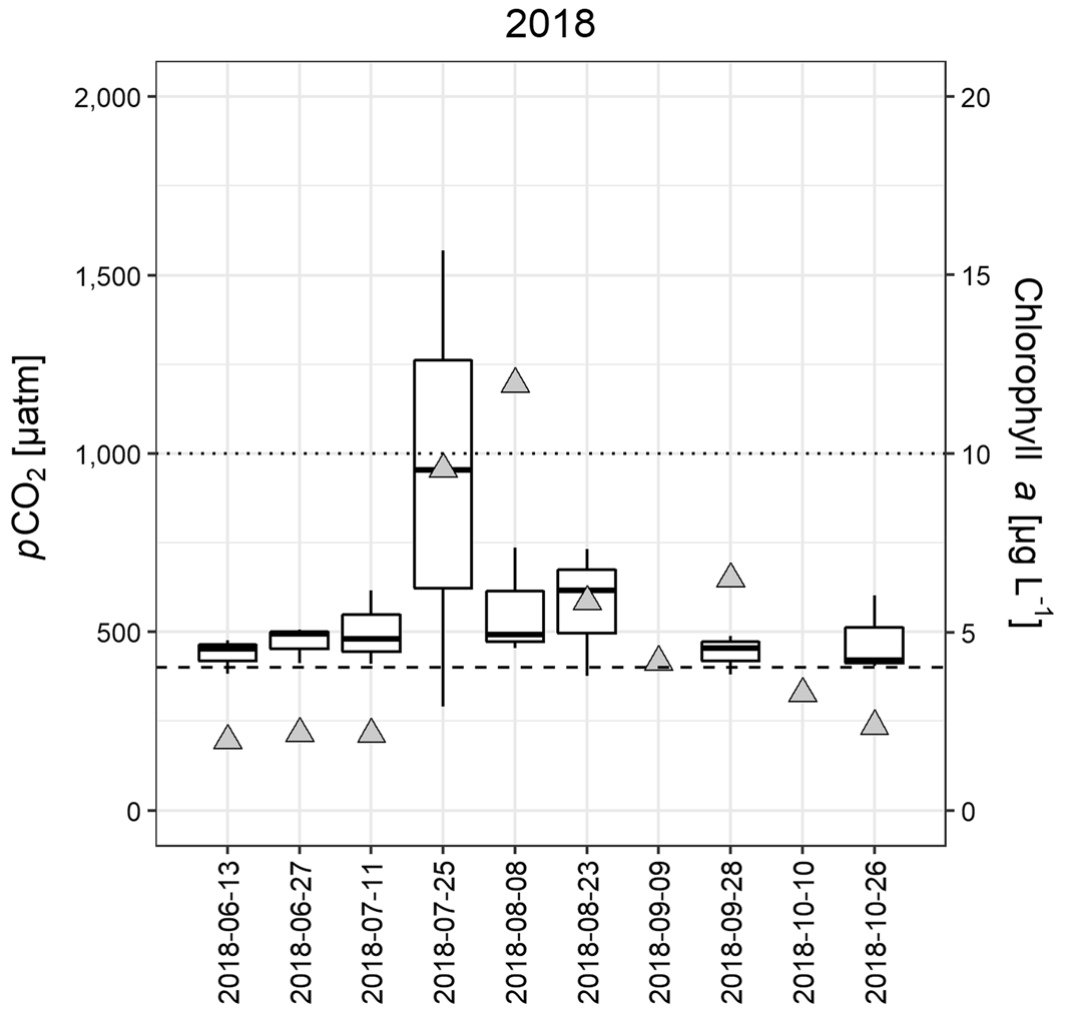
Lake *p*CO_2_ and chlorophyll *a* concentration in Erken in 2018. For each date in the figure, results from 3 sampling locations (*p*CO_2_ measurements, 4 replicates per sampling spot) or one sampling location (chlorophyll *a*) are shown. The boxes symbolize 50% of the data and the line is the median. Chlorophyll *a* concentration was measured from an integrated water sample of either the epilimnion (2018–06-13 until 2018–09-09) or the whole water column after autumn stratification started (2018–09-28 – 2018–10-26). Boxplots = *p*CO_2_, grey triangles = chlorophyll *a*, dashed line = *p*CO_2_ equilibrium (400 µatm), dotted line = chlorophyll *a* threshold indicating an algal bloom (10 µg L^−1^).

We confirmed the presence of the bloom-forming raphidophyte *G. semen* in Erssjön in all DNA samples (analysis: qPCR) taken in 2017 and 2018. Densities of *G. semen* (Fig. 1) varied from being almost absent (110 and 20 cells L^−1^ in 2017 and 2018 respectively) to bloom conditions (102,760–119,660 cells L^−1^ in May, June and August 2017, Fig. 1a), based on our phytoplankton bloom identification of cell abundances > 100,000 cells L^−1^^23^. In 2018, no *G. semen* bloom was observed (maximum abundance 20,360 cells L^−1^ in 2018–05-31, Fig. 1b). In Erken, the bloom-forming cyanobacterium *G. echinulata* was present in phytoplankton samples from 2018–07-17 to 2018–08-14. Chlorophyll *a* concentration in 2018 ranged from 1.9 – 11.95 µg L^−1^ (Fig. 2) and was only once high enough to be considered as a potential phytoplankton bloom according to EU-monitoring standards^23^ (11.95 µg L^−1^, 2018–08-08). At this date, *G. echinulata* densities and biomass was high enough to be considered a bloom (81,588 cells L^−1^, biomass: 0.37 mm^3^ L^−1^) based on WHO guidelines^24^ (2000 cells mL^−1^ or 0.2 mm^3^ L^−3^).

In Erssjön we found a typical summer phytoplankton community for a humic, mesotrophic lake (based on four sampling occasions in 2018). Dominant phytoplankton groups were green algae, cryptophytes and colonial cyanobacteria (20–45% of the total phytoplankton biomass). Phytoplankton biomass was high (8.77–13.54 mm^3^ L^−1^), with *G. semen* making up for 0.5–10% of the total phytoplankton community biomass (Supplementary material, Table S1). The number of species ranged from 24 to 30 and Shannon diversity (H’) ranged from 0.5 to 1.1. No single species dominated the phytoplankton community during this time. The C_phyto_:TOC ratio (= phytoplankton share in total organic carbon) in summer 2018 ranged from 5–9%.

The phytoplankton community in Erken from June to October in 2018 was mainly comprised of diatoms, cryptophytes, dinoflagellates and cyanobacteria. Cryptophytes and dinoflagellates dominated the community in June and July, while cyanobacteria took over in August. Once autumn mixing started in mid-September, diatoms dominated the phytoplankton community up to 79% (2018–10-23, Supplementary material, Table S2). Total phytoplankton biomass (maximum: 2.9 mm^3^ L^−1^) was 3–6 times lower compared to Erssjön. The number of species ranged from 20 to 43 and Shannon diversity was (H’) ranged from 0.47 to 0.92. The C_phyto_:TOC ratio remained < 5% through all seasons.

### pCO_*2*_ and its relation to phytoplankton blooms

Lake *p*CO_2_ in Erssjön ranged from 334 to 1737 µatm in 2017 (Fig. 1a) and from 527 to 1290 µatm in 2018 (Fig. 1b). Thus, Erssjön was supersaturated in CO_2_ (*p*CO_2_ > 400 µatm) during the sampling period, except for 2017–05-16 (Fig. 1a). In 2017, *p*CO_2_ decreased during spring, increased at the beginning of summer and then once at the beginning of autumn. In 2018, there was a different seasonal pattern: *p*CO_2_ remained constant during late spring and early summer, and started to increase from the middle of summer onwards. In early autumn, *p*CO_2_ decreased before increasing again in the middle of autumn. There was no significant relationship between *G. semen* densities and *p*CO_2_ in 2017 (linear regression, *p* = 0.52, R^2^ = 0, n = 29). In 2018, *p*CO_2_ did decrease slightly with increasing *G. semen* abundance (linear regression, R^2^ = 0.15, *p* = 0.02, n = 32).

Lake *p*CO_2_ in Erken ranged from 291 to 1569 µatm in summer and autumn of 2018, with most occasions showing a slight supersaturation (450 to 550 µatm, Fig. 2). The highest *p*CO_2_ was observed in the end of July (2018–07-25), though *p*CO_2_ varied considerably between replicates at those dates. Overall, there was no significant fluctuation in *p*CO_2_ between samplings (Kruskal–Wallis test, chi-squared = 3.51, df = 7, *p* = 0.84). While the second highest chlorophyll *a* concentration (9.56 µg L^−1^) was observed during the *p*CO_2_ maximum (2018–07-25), there was no significant relationship between chlorophyll *a* concentration and *p*CO_2_ (linear regression, R^2^ = 0.1, *p* = 0.13, n = 23) over the year. Phytoplankton total biomass and *p*CO_2_ (linear regression, R^2^ < 0.01, *p* = 0.7, n = 23) and total phytoplankton density and *p*CO_2_ (linear regression, R^2^ < 0.01, *p* = 0.4, n = 23) were not significantly correlated with each other either.

**Figure 3:**
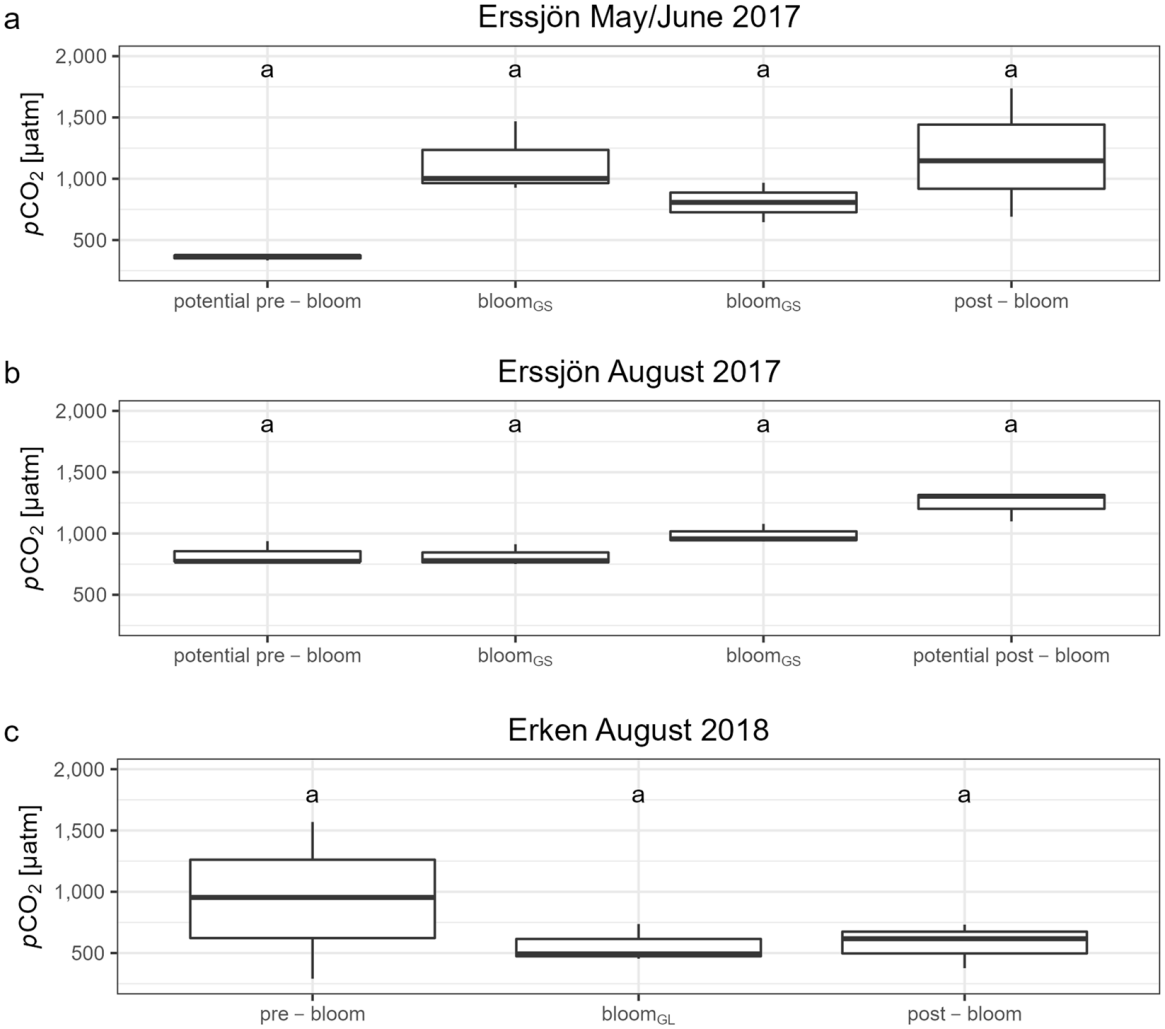
Lake *p*CO_2_ before, during and after observed and potential blooms of *G. semen* (Erssjön) and *G. echinulata* (Erken). In May 2017 in Erssjön, no *G. semen* density data is available for the potential pre- and post-bloom periods. Letters signify the results of the post-hoc Test (Dunn-test, *p* values adjusted with the Bonferroni method) for the Kruskal–Wallis test results. bloom^GS^ = *G. semen* bloom, bloom^GL^ = *G. echinulata* bloom.

Since a relationship between phytoplankton and *p*CO_2_ might be non-linear, we also used an additional approach where we took a closer look at the three periods when algal blooms occurred: May/June and August 2017 (Erssjön) and August 2018 (Erken). Using the Kruskal–Wallis test, we compared *p*CO_2_ during bloom periods (*G. semen* abundance and chlorophyll *a* maximum respectively) to *p*CO_2_ sampled two weeks before (= pre-bloom) and after (= post-bloom) the blooms were detected. Applying this approach for Erssjön, we found that *p*CO_2_ was similar before, during and after the *G. semen* bloom in May/June 2017 (Kruskal–Wallis test, chi-squared = 7.62, df = 3, *p* value = 0.06, Fig. 3a). During the bloom in August 2017, *p*CO_2_ appeared to be significantly higher after the *G. semen* bloom compared to before and during the bloom was observed (Kruskal–Wallis test, chi-squared = 8.74, df = 3, *p* value = 0.03, Fig. 3b). However, the post-hoc test revealed that *p*CO_2_ during this bloom was no different from before and after the bloom (Dunn-test with Bonferroni correction for *p* values, all *p* values > 0.05, Fig. 3b), indicating that the significant result from the Kruskal–Wallis test was due to a type-I error.

Also, in Erken, *p*CO_2_ remained the same before, during and after the *G. echinulata* bloom in August 2018 (Kruskal–Wallis test, chi-squared = 0.62, df = 2, *p* value = 0.72, Fig. 3c). Since chlorophyll *a* concentration was quite high two weeks before the *G. echinulata* bloom (2018–07-25, “pre-bloom” in Fig. 3c), we also ran a Kruskal–Wallis test including 2018–07-11 as the pre-bloom period, to account for either a longer bloom period or two separate blooms happening on 2018–07-11 and 2018–07-25. Still, neither the pre-bloom nor the post-bloom *p*CO_2_ was higher or lower than *p*CO_2_ during the blooms (Kruskal–Wallis test, chi-squared = 1.05, df = 3, *p* value = 0.79).

### pCO_*2*_ and its relation physical lake variables

During sampling, water temperatures at 1-m depth in Erssjön ranged from 5 to 20 °C in 2017 and from 5 to 24 °C in 2018, with summer temperatures (June – August) > 16 °C (Supplementary material, Table S3). Air temperatures ranged from − 1 to 21 °C (2017) and − 1 to 28 °C (2018). Wind speed ranged from 0.2 to 8.4 m^1^ s^−1^ (2017) and 0.2 to 6.4 m^1^ s^−1^ (2018), the average wind speeds being 2.3 and 2.4 m^1^ s^−1^ for 2017 and 2018 respectively. In Erken, water temperatures ranged from 6 to 24 °C (18 – 24 °C between June–August), air temperatures from 2 to 27 °C and wind speed from 0.4 to 8.3 m^1^ s^−1^ (average: 3.7 m^1^ s^−1^).

We observed no significant correlations between *p*CO_2_ and any of the described above physical variables in the two lakes (Supplementary material, Table S4). In 2018, there was a trend towards decreasing *p*CO_2_ with increasing wind speed in Erssjön, but very little of the overall variation in *p*CO_2_ was explained by this (R^2^ = 0.06). Furthermore, water column stability (expressed as the Schmidt number) and gas transfer velocity for water–air gas exchange (expressed as k_600_) were not significantly correlated with *p*CO_2_ either.

## Discussion

In this study we investigated whether the occurrence of phytoplankton blooms can suppress *p*CO_2_ in lakes. We studied two different lakes, one in which cyanobacterial (*G. echinulata*) blooms occurred (Erken), and another one in which we observed blooms of the raphidophyte *G. semen* (Erssjön)*.* Contrary to what we expected the phytoplankton signal was not detectable as short-term fluctuations in *p*CO_2_ during those times, which indicates that other factors are more important for *p*CO_2_ in those lakes.

One criterium for phytoplankton to impact lake *p*CO_2_ is a high enough biomass so that photosynthesis dominates over bacterial respiration and mineralization of carbon^10^. Available C_phyto_:TOC ratios were > 5% in Erssjön during summer of 2018. Since this is a threshold value for when phytoplankton biomass potentially can have a detectable effect on CO_2_^10^, we would have expected that a phytoplankton bloom in this lake caused a decrease in *p*CO_2_. Since our hypothesis could not be supported, an interesting question in this context is the identity of the bloom forming phytoplankton species, especially the proportion of mixotrophic species. Erssjön’s phytoplankton community is typical for a boreal brown water lake with a low pH, with cryptophytes, chrysophytes, euglenids and chlorophytes dominating in the summer of 2018. Some of these are mixotrophic species (*Uroglena* sp., *Dinobryon* sp., *Gymnodium* sp., *Perdinium* sp. and *Ceratium* sp.^25^, see Supplementary material, Table S5) and could potentially significantly contribute to heterotrophic respiration instead of autotrophic carbon fixation in boreal lakes^26^, especially in a warming climate^27^. This could be one contributing factor as to why CO_2_ in many boreal brown water lakes is seemingly not impacted by photosynthetic processes and remains supersaturated throughout the year, even though theses lakes demonstrate a high phytoplankton biomass with bloom conditions.

In Erken, the total phytoplankton biomass remained relatively low (< 2 mm^3^ L^−1^) during most of the year, which is below the C_phyto_:TOC ratio of 5% that Engel et al.^10^ identified as a threshold for a detectable phytoplankton influence on lake CO_2_. However, a phytoplankton bloom still occurred (*G. echinulata* in August), where also chlorophyll *a* concentrations were higher than 10 µg L^−1^ (total phytoplankton biomass: 1.12 mm^3^ L^−1^). However, again, these bloom conditions did not leave a detectable signal on the *p*CO_2_. The lack of a direct relation between phytoplankton and *p*CO_2_ in Erken might be a result of measuring phytoplankton and *p*CO_2_ at different sites. Erken is a relatively big lake that is strongly wind exposed with frequent lake water mixing, causing a patchy phytoplankton distribution, especially regarding large floating *G. echinulata* colonies.

Wind exposure is not only affecting the phytoplankton distribution in a lake but also* p*CO_2_. Previous studies have shown that wind-induced upwelling and vertical mixing affect the transport of CO_2_ through the water column^28,29^, as well as the CO_2_ flux to the atmosphere^30^. It is possible that wind suppresses the phytoplankton signal on lake *p*CO_2_, because CO_2_ at the lake surface, where phytoplankton are photosynthesizing and, thus, consuming CO_2_, is mixed with CO_2_ from waters from below. This may be especially true at Erken, which is highly susceptible to wind mixing, even causing perturbations in the thermal stratification during summer^31^. Furthermore, wind exposure may explain why lake *p*CO_2_ does not increase in autumn after mixing starts in this lake, even though this is typically observed in eutrophic lakes when deep-water, CO_2_ water mixes with surface oxygen-rich water^32^.

However, the *G. echinulata* bloom in Erken occurred mostly at low wind speeds (1.2–5.4 m^1^ s^−1^, mean: 2.9 m^1^ s^−1^) when water mixing is low, as did the *G. semen* blooms in Erssjön (0.5–5.1 and 0.4 – 2.8 m^1^ s^−1^ in May and June respectively). Consequently, CO_2_ import from deeper waters should be low. On the other hand, the gas transfer velocity of CO_2_ from water to the atmosphere is lower at low wind speeds, which could lead to higher observed *p*CO_2_ than would be expected during periods of high photosynthetic activity. Thus, the interaction between wind-controlled processes in the water column may be why *p*CO_2_ and physical lake variables do not correlate in our study.

Besides mixing, inorganic carbon import via groundwater and river inflow could have affected *p*CO_2_ in both lakes, which is common for lakes in the studied geographical region^3^. This could for example explain the increase of *p*CO_2_ in Erssjön in autumn in 2017 and 2018, as this is when autumn mixing starts, precipitation events become more common and water in- and outflow from the lake increase. Furthermore, Erssjön is a rather small lake with a potentially shorter water retention time than Erken (7 years), so hydrological factors contributing to CO_2_ supersaturation might be dominating over biological processes.

Even if blooms caused by *G. semen* or other phytoplankton species lead to a decrease in lake *p*CO_2_, it is unclear whether this would have a short-term or long-term effect on lake CO_2_ emissions. Autochthonous carbon is preferably used by bacteria and often gets re-mineralized before it can sink to the lake bottom and contribute to the fixed carbon in the sediment^33,34^. Erssjön’s *p*CO_2_ increased in autumn in 2017 and 2018, indicating that phytoplankton biomass may contribute to internal lake carbon respiration and thus CO_2_ emissions from the lake when being degraded. In order to understand the ecosystem effects, we would therefore need to monitor *G. semen* blooms and phytoplankton in general more closely, both regarding their biomass and the sedimentation of organic material before, during and after a bloom.

Sampling frequency is also critical for capturing times when *G. semen* migrates vertically through the water column during the day^35^. During migration, it easy to underestimate *G. semen* densities by sampling too early or too late during the day. It is also known that the carbon flux has high diel and seasonal variability^32,36^, with diel variations being especially pronounced during mixing events^32^. Even though the floating chambers were deployed for around 24 h, deployment and measurement times were not consistent between samplings (ranging from ~ 8:00 to 20:00, see Supplementary material, Table S6). This means that changes in *p*CO_2_ during the day may not always have been captured completely. Thus, it is likely that the *p*CO_2_ measurements at the surface of the lakes caused an under- or overestimation of *p*CO_2_ for our sampling dates due to the time of sampling.

In summary, our results show that neither *G. semen* nor *G. echinulata* blooms were associated with decreases in *p*CO_2_ in two Swedish boreal lakes. It is likely that physical factors, such as wind induced water column mixing and import of inorganic carbon via groundwater inflow and runoff, suppress the phytoplankton signal on *p*CO_2_. A higher sampling frequency is necessary to further study the dynamics between phytoplankton blooms and lake CO_2_ concentrations, as phytoplankton blooms can occur rapidly and disappear within days. Further studies should focus on monitoring phytoplankton biomass and *p*CO_2_ at higher frequency to capture diel variations, and include sedimentation rates of autochthonous organic matter and daily CO_2_ fluctuations to determine if there is a long-term effect of phytoplankton on lake carbon fluxes. Another interesting aspect to study is the species composition of phytoplankton blooms, since the degree of mixotrophy could affect the relationship between phytoplankton and *p*CO_2_.

## Methods

### Sampling sites

Sampling was performed in a mesotrophic brown water lake (Erssjön) in the Bäveån catchment in Västergötland, Sweden (58°22′16.5"N, 12°09′40.3"E) and in a mesotrophic non-humic lake (Erken) in the Broströmmen catchment in Uppland, Sweden (59°50′26.35"N, 18°37′39.75"E). Background information about the two lakes are publicly available at the SITES website (https://www.fieldsites.se/en-GB/sites-thematic-programs/lakes-in-sites-44856978). Erssjön is situated 73 m above sea level, has an area of 0.061 km^2^ and a maximum depth of 4.5–5 m. The surrounding area is mostly forest and some agricultural land. The lake is dimictic, with anoxic periods during summer stratification. Samples were taken from March to October in 2017 (16 CO_2_ samplings, 11 DNA samplings) and from May to November in 2018 (13 CO_2_ samplings, 11 DNA samplings) every 2–4 weeks.

Erken is 13 m above sea level, has an area of 24 km^2^ and a maximum depth of 21 m. The surrounding area is mostly forest, water and some agricultural land and pasture. The lake is dimictic. Samples were taken from June to October in 2018 (8 CO_2_ samplings, every 2–4 weeks). Phytoplankton densities and chlorophyll *a* are monitored through weekly samplings conducted by the Erken Laboratory.

### *CO*_*2*_ measurements

**Figure 4:**
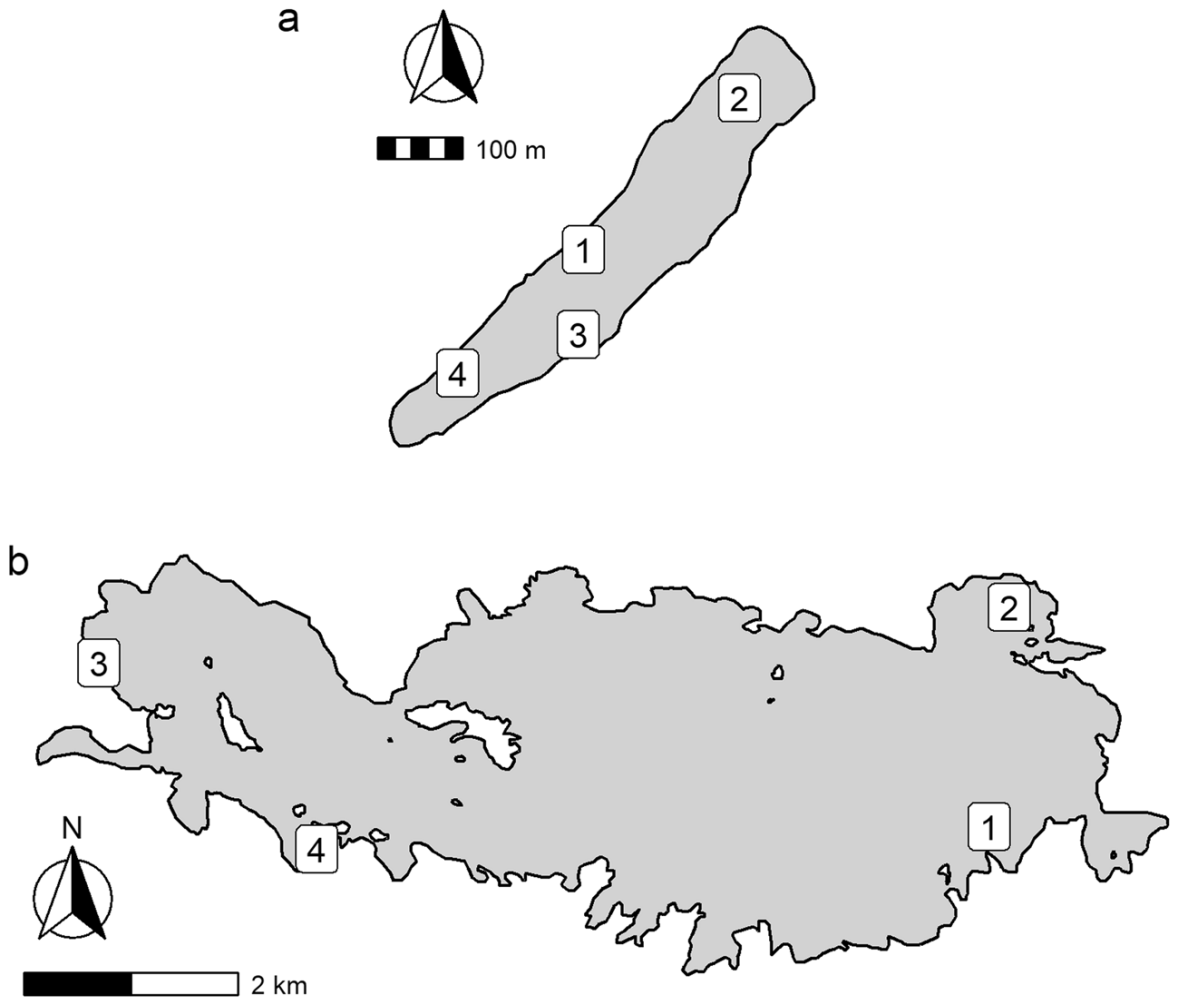
Floating chamber and phytoplankton sampling locations in Erssjön (**a**) and Erken (**b**). Numbers in Erssjön (**a**) refer to 1 = phytoplankton and DNA sampling location, 2 = chambers A1 – A4, 3 = chambers B5 – B8, 4 = chambers C9 – C12, numbers in Erken (**b**) to 1 = phytoplankton and chlorophyll sampling location, 2 = NJÄ_01 – NJÄ_04, 3 = KHM_01 – KHM_04, 4 = SBA_01 – SBA_04. For individual chamber coordinates, refer to Supplementary material, Table S7. This map was generated with the R software (R 4.1.2, package: ggmap, ggspatial) with shapefiles created in Google Earth.

Carbon dioxide concentrations were measured with floating chambers designed according to Bastviken et al.^37^. In each lake, 4 floating chambers were set along 3 transects from the lake shore towards the center (Fig. 4, check Supplementary material, Table S7 for coordinates). The lake water depths below the floating chambers ranged from 0.3 to 1.9 m (Erssjön) and 0.6 – 4.7 m (Erken). After a deployment of 18–28 h (Supplementary material, Table S6) a subsample of the headspace was taken with a syringe (3 × 60 mL) and transferred into a gas-tight, sealed glass vial through a needle (0.5 mm; 25 gauge). Samples were measured on a gas chromatograph autosampler. These headspace CO_2_ concentrations should be close to equilibrium and *p*CO_2_ was calculated according to Henry’s Law. This data is publicly available upon request on the SITES website (www.fieldsites.se).

### Phytoplankton bloom identification

Phytoplankton blooms in lake surface waters were identified by analyzing chlorophyll *a* concentration, phytoplankton biomass and phytoplankton densities^38,39^. Sampling for those variables took place between 9:00 and 12:00 at the two lakes. A chlorophyll *a* threshold of 10 µg L^−1^ is often used to indicate a phytoplankton bloom^23,40^. In addition, phytoplankton biomass is often monitored to identify blooms of harmful algal species (HABs), e.g. toxic cyanobacteria^40,41^. To identify phytoplankton blooms in the two study lakes, we decided to use a chlorophyll *a* threshold of 10 µg L^−1^ to screen for phytoplankton blooms, as is recommended for moderately deep lakes in Europe^23^. For Erken, chlorophyll *a* data was available from the weekly monitoring program upon request (Erken Laboratory, Uppsala University, Sweden, https://www.ieg.uu.se/erken-laboratory/lake-monitoring-programme/), and is now also available on the SITES Data portal:

(https://meta.fieldsites.se/objects/NT59BPT70Ce7h1huzA0xSc1c). For chlorophyll analysis, water was filtered through a Whatman GF/C filter. Chlorophyll was extracted with 90% acetone and absorbance measured with a spectrophotometer at 750, 664, 647 and 630 nm according to the Swedish standard SS028146.

Since reliable chlorophyll *a* data from Erssjön was not available, we also identified blooms by determining densities of *G. semen* (qPCR) and *G. echinulata* (phytoplankton monitoring data). For densities the following thresholds for a bloom identification were used: 2,000 cells mL^−1^ or 0.2 mm^3^ L^−1^ for *G. echinulata* which is the lowest WHO threshold for dangerous cyanobacteria blooms^40^, based on the fact that these species are potentially toxic^42,43^, and 100,000 cells L^−1^ for *G. semen* since this species has an unusual big cell size^44^.

### DNA analysis for the identification of G. semen densities

In Erssjön, a DNA sample was taken every 2–4 weeks from the Southern side of the mesocosm platform (N 58.371322°, E 12.161074°, see Fig. 4a) during 2017–05-15 – 2017–10-16 and 2018–05-15 – 2018–11-01. An integrated water sample (0–1 m) of 10 L was taken and a subsample of 2 L filtered through a membrane filter (0.2 µm pore size, 47 mm diameter). The filter was then folded, put into a 2 mL cryotube and stored at − 80 °C. DNA was extracted with the Qiagen DNeasy PowerSoil Kit according to the kit’s instruction manual. The DNA concentrations were quantified using PicoGreen, measured on a Tecan Spark plate reader (Tecan, Switzerland) and found to be high enough for further analysis (~ 3–20 µg DNA µL^−1^).

For determining the *G. semen* abundance, we used qPCR with the specific primers developed by Johansson et al.^45^. The DNA was diluted 20 × and the master mix done with SYBR Green. The qPCR was run on a CFX96 Touch Real-Time PCR Detection System (Bio-Rad, USA) with the following cycle conditions: 2 min at 90 °C, 40 cycles at 95 °C for 15 s, 40 cycles at 60 °C for 15 s and 40 cycles at 72 °C for 15 s. For each sample, three technical replicates were measured and the average copy number per vegetative cell was used for quantification of *G. semen* cells. To determine the copy number per vegetative cell, we measured the extracted and 10 × diluted DNA of three samples of isolated and washed *G. semen* vegetative cells (40 cells per sample) from a *G. semen* culture established from a lake in Northern Uppland (Siggefora, culture SF1A8, established by Ingrid Sassenhagen). More details on this procedure is available in Münzner et al.^46^. The copy number per cell ranged from 171,500 to 304,770, with an average copy number of 243,596 (standard deviation: 54,605) per *G. semen* vegetative cell.

### Phytoplankton community composition

To get a better understanding on phytoplankton dynamics we also determined the phytoplankton biomass and community composition. In Erssjön we sampled during four dates in 2018 (July 10, July 24, August 14, September 1). Phytoplankton samples were taken from surface water samples (~ 0.5 m depth) and a subsample 100 mL was fixed with Lugol. Samples were stored at 4 °C in the dark before analysis. Phytoplankton samples were acclimatized to room temperature (24 h) and were counted with the Utermöhl method with an inverted microscope (Leica DMIL LED fluo) after settling for at least 12 h in a 10 mL chamber. We did a whole chamber count at 40 × magnification for phytoplankton > 100 µm (e.g. colonial algae), counts of diagonals (1 or 2) at 200 × magnification until a cell count of at least 200 cells (> 20 µm) was reached, and 10 random field counts at 400 × magnification. For each identified species, cell length and width were measured for one individual and used for biomass calculations. Phytoplankton shapes and biovolume formulas were determined according to Hillebrand et al.^47^. Phytoplankton diversity (H’) and evenness (e_h_) were calculated using the Shannon–Wiener Index.

For Erken, we used publicly available plankton data from the Erken Laboratory (Uppsala University, Sweden, https://www.ieg.uu.se/erken-laboratory/lake-monitoring-programme/, data available upon request). Phytoplankton samples used in this data set were collected the following way: phytoplankton samples were collected at the deepest point of the lake with an integrated water sampler (“Ramberg tube”) in 2 m intervals. The samples were mixed together according to their proportion to the total lake volume (all samples if the lake was fully mixed, epilimnion and hypolimnion samples mixed separately if the lake was stratified). Then, a 100 mL subsample of the integrated sample (only epilimnion sample if the lake was stratified) was taken, fixated with a few drops of Lugol and stored at 4 °C before further analysis. Phytoplankton were identified and quantified with an inverted light microscope according to the quality standards (SS-EN 15,204:2006) approved by the Swedish Environmental Protection Agency (EPA).

From the phytoplankton data we calculated the C_phyto_:TOC ratio to identify a previously described threshold when phytoplankton dynamics become detectable in lake waters^10^. For C_phyto_ a conversion factor of 0.15 was chosen according to Engel et al.^10^. For TOC (total organic carbon) we used data on dissolved organic carbon (DOC). DOC has been shown to stand for 97% of the TOC in boreal lakes^48^, thus we assumed DOC to be equal to TOC in our study lakes. Since carbon monitoring data were not available for Erssjön, we used an average of previously reported values (22.33 mg DOC L^−1^) by Groeneveld et al.^49^.

### Lake physical variables

Publicly available data collected by SITES (SITES Data portal: https://data.fieldsites.se/portal/) of air temperature, water temperature at 1-m depth, wind direction and wind speed were used. For analyses, average values of those variables over the total deployment time of the chambers at each date were calculated. Schmidt number Sc was calculated according to Wanninkhof et al.^50^, and for piston velocity k_600_, the formula for low wind speeds by Cole and Caraco^51^ was used.

### Statistics

All data were analyzed with R 4.1.2. Group comparisons of CO_2_ measurements within transects with the Kruskal–Wallis test (post-hoc test: Dunn-test with Bonferroni correction for *p* values) showed that *p*CO_2_ was significantly higher in the chambers closest to the shore compared to the chambers further away from the shore, both in Erssjön (chi-square = 9.18, df = 3, *p* = 0.03) and Erken (chi-square = 8.43, df = 3, *p* = 0.04). However, even though *p*CO_2_ decreased with increasing distance to the shore (linear regression analysis) in Erssjön (*p* < 0.001, R^2^ = 0.02, n = 316) and Erken (*p* < 0.05, R^2^ = 0.05, n = 104), almost none of the overall variation in *p*CO_2_ was explained by that. Following this, we used the average *p*CO_2_ of each transect for further analyses (n = 3 per lake per sampling).

Shown regressions and correlations are based on linear relationships. Since the distribution of all variables was skewed, they were log-transformed. Changes in *p*CO_2_ over sampling dates in each lake for each year were assessed with the Kruskal–Wallis test. The Kruskal–Wallis test was also used for comparisons of *p*CO2 before, during and after phytoplankton blooms. For post-hoc analysis of significant results, the Dunn test was used and *p* values were adjusted with the Bonferroni method (package: FSA).

### Supplementary Information

Supplementary Information.

## Data Availability

The datasets generated during and/or analysed during the current study are available in the DiVA (Digitala Vetenskapliga Arkivet) portal, [urn:nbn:se:uu:diva-479668].
