# Supplementary material for: Carbon dioxide reduction by photosynthesis undetectable even during phytoplankton blooms in two lakes
Source: Sci Rep. 2023 Aug 19;13:13503. doi: 10.1038/s41598-023-40596-6 (PMC10439937; doi:10.1038/s41598-023-40596-6)
Supplement: Supplementary file 1 — Supplementary Information. [file 41598_2023_40596_MOESM1_ESM.docx]

**Supplementary material Table S1:** Characteristics of the phytoplankton community in Erssjön from 2018-07-10 to 2018-09-01. C_(phyto)_ = Phytoplankton carbon, calculated as phytoplankton biomass multiplied by a conversion factor of 0.15 (Engel et al. 2019), TOC = total organic carbon, C_(phyto)_/TOC ratio = phytoplankton share in total organic carbon. In lakes where C_(phyto)_/TOC ratio >5, phytoplankton activity can potentially reduce *p*CO_2_ (Engel et al. 2019).

|  | **2018-07-10** | **2018-07-24** | **2018-08-14** | **2018-09-01** |
| --- | --- | --- | --- | --- |
| Total phytoplankton biomass [mm^3^ L^-1^] | 12.18 | 6.90 | 8.06 | 10.89 |
| Number of species (=species richness) | 24 | 25 | 30 | 29 |
| Shannon diversity | 0.61 | 1.12 | 0.49 | 0.53 |
| *G. semen* biomass [mm^3^ L^-1^] | 1.46 | 0.39 | 0.03 | 0.09 |
| *G. semen* biomass compared to total phytoplankton biomass [%] | 11.96 | 5.75 | 0.41 | 0.86 |
| Biomass of the dominant species compared to total phytoplankton biomass [%] | 45.22 | 20.91 | 21.12 | 36.11 |
| C_(phyto)_ | 2.05 | 1.64 | 1.32 | 1.72 |
| C_(phyto)_/TOC ratio | 9.16 | 7.35 | 5.89 | 7.68 |

**Table S2:** Characteristics of the phytoplankton community in Erken from 2018-06-12 to 2018-10-23. C_(phyto)_ = Phytoplankton carbon, calculated as phytoplankton biomass multiplied by a conversion factor of 0.15 (Engel et al. 2019), TOC = total organic carbon, C_(phyto)_/TOC ratio = importance of phytoplankton CO2 uptake compared to CO2 production by mineralization. In lakes C_(phyto)_/TOC >5, phytoplankton activity can potentially reduce *p*CO_2_ (Engel et al. 2019).

| **Date** | **total phytoplankton biovolume [mm^3^ L^-1^]** | **Number of species (=species richness)** | **Shannon diversity [H']** | **Species with the highest biomass** | **Biomass of the dominant species compared to total phytoplankton biomass [%]** | **C_(phyto)_** | **C_(phyto)_:TOC ratio** |
| --- | --- | --- | --- | --- | --- | --- | --- |
| 2018-06-12 | 0.28 | 20 | 0.62 | *Plagioselmis nannoplanctica* | 38.00 | 0.04 | 0.36 |
| 2018-06-28 | 0.45 | 31 | 0.47 | *Plagioselmis nannoplanctica* | 22.36 | 0.07 | 0.62 |
| 2018-07-10 | 0.34 | 24 | 0.64 | *Ceratium hirundinella* | 30.92 | 0.05 | 0.45 |
| 2018-07-24 | 0.56 | 32 | 0.85 | *Peridinium* sp. | 12.25 | 0.08 | 0.79 |
| 2018-07-31 | 0.88 | 47 | 0.69 | *Fragilaria crotonensis* | 13.25 | 0.13 | 1.27 |
| 2018-08-07 | 1.12 | 43 | 0.59 | *Gloeotrichia* *echinulata* | 32.95 | 0.17 | 1.48 |
| 2018-08-22 | 1.09 | 36 | 0.93 | *Fragilaria crotonensis* | 36.14 | 0.16 | 1.34 |
| 2018-09-11 | 0.46 | 39 | 0.86 | *Aulacoseira* sp. | 14.13 | 0.07 | 0.64 |
| 2018-09-25 | 2.90 | 30 | 0.79 | *Aulacoseira* sp. | 45.95 | 0.44 | 3.92 |
| 2018-10-09 | 1.13 | 28 | 0.83 | *Aulacoseira* sp. | 56.76 | 0.17 | 1.65 |
| 2018-10-23 | 1.01 | 22 | 0.59 | *Aulacoseira* sp. | 78.77 | 0.15 | 1.36 |

**Table S3**: Lake physical variables at Erken (2018) and Erssjön (2017 and 2018). Air temperature, water temperature at 1-meter depth, wind direction and wind speed are average values over the total deployment time of the chambers at each date. Wind direction is given in degrees clockwise through 360 degrees. The Schmidt number Sc was calculated according to Wanninkhof et al. (1992), and for piston velocity k_600_, the formula for low wind speeds by Cole and Caraco (1998) was used. * = algal bloom observed.

| **lake** | **measurement** | **air temperature** | **water temperature** | **wind direction** | **wind speed** | **Schmidt number** | **piston velocity** |
| --- | --- | --- | --- | --- | --- | --- | --- |
|  | **date** | **[°C] at 1m** | **[°C] at 1m** | **[degrees]** | **[m^-1^ s^-1^]** | **Sc** | **k_600_** |
| Erken | 2018-06-13 | 14.1 | 18.4 | 151 | 2.3 | 651 | 0.68 |
| Erken | 2018-06-27 | 16.3 | 16.9 | 158 | 2.3 | 706 | 0.65 |
| Erken | 2018-07-11 | 18.9 | 18.8 | 135 | 2.9 | 637 | 0.78 |
| Erken | 2018-07-25 | 23.4 | 23.9 | 162 | 3.5 | 497 | 1.02 |
| Erken* | 2018-08-08 | 21.7 | 23.8 | 194 | 2.9 | 500 | 0.89 |
| Erken | 2018-08-23 | 19.5 | 19.8 | 226 | 6.0 | 607 | 1.59 |
| Erken | 2018-09-28 | 8.2 | 13.4 | 219 | 5.1 | 848 | 1.12 |
| Erken | 2018-10-26 | 4.7 | 9.6 | 323 | 4.7 | 1059 | 0.92 |
| Erssjön | 2017-03-28 | 4.7 | 6.3 | 144 | 2.5 | 1291 | 0.51 |
| Erssjön | 2017-04-12 | 6.6 | NA | 232 | 3.1 | NA | NA |
| Erssjön | 2017-05-05 | 7.3 | 11.1 | 70 | 3.2 | 972 | 0.68 |
| Erssjön | 2017-05-16 | 10.3 | 12.3 | 139 | 1.4 | 903 | 0.48 |
| Erssjön* | 2017-05-30 | 13.0 | 18.2 | 229 | 1.9 | 656 | 0.62 |
| Erssjön* | 2017-06-13 | 14.7 | 16.1 | 236 | 3.0 | 734 | 0.74 |
| Erssjön | 2017-06-27 | 13.6 | 18.2 | 171 | 2.5 | 657 | 0.71 |
| Erssjön | 2017-07-11 | 16.4 | 19.7 | 226 | 1.5 | 609 | 0.59 |
| Erssjön | 2017-07-25 | 15.9 | NA | 95 | 2.7 | NA | NA |
| Erssjön | 2017-08-08 | 15.3 | 18.3 | 207 | 2.0 | 654 | 0.63 |
| Erssjön* | 2017-08-22 | 13.5 | 18.0 | 143 | 1.5 | 662 | 0.58 |
| Erssjön* | 2017-09-05 | 13.1 | 15.8 | 45 | 2.2 | 746 | 0.63 |
| Erssjön | 2017-09-21 | 10.1 | 13.0 | 47 | 2.6 | 870 | 0.63 |
| Erssjön | 2017-10-03 | 12.0 | 12.1 | 224 | 3.1 | 916 | 0.69 |
| Erssjön | 2017-10-17 | 12.1 | 10.9 | 228 | 2.6 | 981 | 0.6 |
| Erssjön | 2017-10-31 | 0.4 | 5.6 | 217 | 1.1 | 1350 | 0.37 |
| Erssjön | 2018-05-18 | 11.5 | 18.2 | 54 | 3.7 | 658 | 0.93 |
| Erssjön | 2018-05-31 | 19.9 | 22.2 | 62 | 2.9 | 540 | 0.85 |
| Erssjön | 2018-06-15 | 13.6 | 19.1 | 232 | 3.9 | 627 | 1 |
| Erssjön | 2018-06-27 | 19.7 | 18.6 | 162 | 1.3 | 644 | 0.55 |
| Erssjön | 2018-07-11 | 19.2 | 22.0 | 95 | 2.3 | 543 | 0.75 |
| Erssjön | 2018-07-25 | 24.2 | 23.5 | NA | NA | 506 | NA |
| Erssjön | 2018-08-15 | 15.9 | 18.8 | NA | NA | 636 | NA |
| Erssjön | 2018-08-31 | 13.9 | 16.6 | 144 | 1.9 | 712 | 0.6 |
| Erssjön | 2018-09-20 | 15.4 | 15.5 | 232 | 4.9 | 757 | 1.13 |
| Erssjön | 2018-10-04 | 3.8 | 9.2 | 70 | 1.1 | 1085 | 0.42 |
| Erssjön | 2018-10-19 | 4.3 | 11.3 | 139 | 0.8 | 956 | 0.42 |
| Erssjön | 2018-11-01 | 4.1 | 4.8 | 229 | 1.0 | 1417 | 0.36 |
| Erssjön | 2018-11-15 | 8.6 | 7.5 | 236 | 2.1 | 1202 | 0.48 |

**Table S4**: results of linear regression analyses between *p*CO_2_ and lake physical variables. Sc = Schmidt number calculated according to Wanninkhof et al. (1992). k_600_ = piston velocity calculated according to Cole and Caraco (1998). All variables were logged before analyses. Numbers denote the R^2^, symbols the p-value. n = number of samples. abbreviations: . = < 0.1, * = < 0.05, ** = < 0.01, *** = < 0.001.

| **Independent** | **Dependent** |  |  |  |  |  |
| --- | --- | --- | --- | --- | --- | --- |
| **variable** | **variable** |  |  |  |  |  |
|  | *p*CO_2_ |  |  |  |  |  |
|  | Erken 2018 |  | Erssjön 2017 |  | Erssjön 2018 |  |
| Air temperature | 0.04 | n = 23 | 0 | n = 47 | 0 | n = 37 |
| Water temperature | 0.05 | n = 23 | 0 | n = 41 | 0 | n = 37 |
| Wind speed | 0 | n = 23 | 0 | n = 47 | 0.06 . | n = 31 |
| Wind direction | 0 | n = 23 | 0 | n = 47 | 0 | n = 31 |
| Sc | 0.07 | n = 23 | 0 | n = 41 | 0 | n = 37 |
| k_600_ | 0 | n = 23 | 0 | n = 41 | 0.09 . | n = 31 |

**Table S5:** Presence of mixotrophic species according to Sanders and Porter [23] in Erssjön from 2018-07-10 to 2018-09-01. x = species present, - = species not present.

|  | **2018-07-10** | **2018-07-24** | **2018-08-14** | **2018-09-01** |
| --- | --- | --- | --- | --- |
| *Uroglena* sp. | - | - | x | - |
| *Dinobryon* sp. | x | x | - | - |
| *Gymnodium* sp. | x | x | - | - |
| *Perdinium* sp. | x | - | x | - |
| *Ceratium* sp. | - | - | x | - |

**Table S6**: Floating chamber deployment time periods and weather conditions at Erken (2018) and Erssjön (2017 and 2018). Time periods are given as a range from minimum to maximum deployment time of the 12 chambers deployed at each date. Wind speed and water temperature at 1-meter depth are average values over the total deployment time of the chambers at each date. h = hours, m = minutes, * = algal bloom observed.

| **lake** | **deployment** |  | **measurement** |  | **Minimum deployment** | **Maximum deployment** | **Wind speed** | **Water temperature** |
| --- | --- | --- | --- | --- | --- | --- | --- | --- |
|  | **date** | **time** | **date** | **time** | **time [h:m]** | **time [h:m]** | **[m^-1^ s^-1^]** | **[°C] at 1m** |
| Erken | 2018-06-12 | 13:27 – 16:06 | 2018-06-13 | 8:54 – 12:37 | 19:25 | 20:47 | 2.3 | 18.4 |
| Erken | 2018-06-26 | 11:46 – 14:13 | 2018-06-27 | 11:05 – 13:36 | 23:17 | 23:34 | 2.3 | 16.9 |
| Erken | 2018-07-10 | 12:20 – 16:13 | 2018-07-11 | 12:09 – 14:30 | 22:10 | 23:49 | 2.9 | 18.8 |
| Erken | 2018-07-24 | 10:32 – 12:28 | 2018-07-25 | 9:42 – 11:53 | 22:38 | 23:39 | 3.5 | 23.9 |
| Erken* | 2018-08-07 | 9:43 – 12:00 | 2018-08-08 | 8:50 – 11:17 | 22:40 | 23:18 | 2.9 | 23.8 |
| Erken | 2018-08-22 | 9:09 – 12:31 | 2018-08-23 | 8:55 – 11:35 | 23:01 | 23:57 | 6.0 | 19.8 |
| Erken | 2018-09-27 | 10:49 – 13:53 | 2018-09-28 | 10:48 – 14:45 | 23:36 | 24:49 | 5.1 | 13.4 |
| Erken | 2018-10-25 | 10:53 – 13:23 | 2018-10-26 | 8:19 – 11:43 | 21:26 | 22:24 | 4.7 | 9.6 |
| Erssjön | 2017-03-27 | 11:50 – 12:28 | 2017-03-28 | 10:09 – 11:06 | 22:19 | 22:41 | 2.5 | 6.3 |
| Erssjön | 2017-04-11 | 14:43 – 15:45 | 2017-04-12 | 14:03 – 14:57 | 23:07 | 23:20 | 3.1 | NA |
| Erssjön* | 2017-05-04 | 11:00 – 11:47 | 2017-05-05 | 10:11 – 10:56 | 22:29 | 23:56 | 3.2 | 11.1 |
| Erssjön | 2017-05-15 | 15:23 – 15:58 | 2017-05-16 | 15:40 – 16:15 | 24:12 | 24:17 | 1.4 | 12.3 |
| Erssjön | 2017-05-29 | 15:07 – 15:45 | 2017-05-30 | 13:59 – 14:33 | 22:44 | 22:52 | 1.9 | 18.2 |
| Erssjön* | 2017-06-12 | 15:47 – 17:42 | 2017-06-13 | 10:49 – 12:52 | 19:02 | 19:23 | 3.0 | 16.1 |
| Erssjön | 2017-06-26 | 15:58 – 16:42 | 2017-06-27 | 11:30 – 12:10 | 19:26 | 19:38 | 2.5 | 18.2 |
| Erssjön | 2017-07-10 | 16:37 – 17:09 | 2017-07-11 | 15:00 – 15:39 | 22:23 | 22:31 | 1.5 | 19.7 |
| Erssjön | 2017-07-24 | 12:56 – 13:27 | 2017-07-25 | 13:08 – 13:46 | 24:11 | 24:19 | 2.7 | NA |
| Erssjön | 2017-08-07 | 9:33 – 10:11 | 2017-08-08 | 9:12 – 9:53 | 23:35 | 23:46 | 2.0 | 18.3 |
| Erssjön | 2017-08-21 | 19:30 – 20:29 | 2017-08-22 | 13:40 – 15:19 | 18:10 | 18:50 | 1.5 | 18.0 |
| Erssjön | 2017-09-04 | 12:09 – 12:41 | 2017-09-05 | 11:56 – 12:36 | 23:21 | 24:37 | 2.2 | 15.8 |
| Erssjön | 2017-09-20 | 13:43 – 14:21 | 2017-09-21 | 11:57 – 12:51 | 22:14 | 22:30 | 2.6 | 13.0 |
| Erssjön | 2017-10-02 | 12:25 – 12:55 | 2017-10-03 | 11:49 – 12:39 | 23:22 | 23:44 | 3.1 | 12.1 |
| Erssjön | 2017-10-16 | 11:35 – 12:19 | 2017-10-17 | 11:21 – 12:14 | 23:46 | 24:00 | 2.6 | 10.9 |
| Erssjön | 2017-10-30 | 14:45 – 15:23 | 2017-10-31 | 11:08 – 11:53 | 20:23 | 20:30 | 1.1 | 5.6 |
| Erssjön | 2018-05-17 | 11:50 – 12:47 | 2018-05-18 | 11:27 – 12:17 | 23:24 | 23:39 | 3.7 | 18.2 |
| Erssjön | 2018-05-30 | 14:56 – 16:05 | 2018-05-31 | 14:19 – 15:17 | 23:08 | 23:23 | 2.9 | 22.2 |
| Erssjön | 2018-06-14 | 15:30 – 16:28 | 2018-06-15 | 13:38 – 14:42 | 22:02 | 22:14 | 3.9 | 19.1 |
| Erssjön | 2018-06-26 | 8:45 – 9:41 | 2018-06-27 | 10:52 – 11:56 | 26:07 | 26:17 | 1.3 | 18.6 |
| Erssjön | 2018-07-10 | 11:39 – 13:06 | 2018-07-11 | 15:31 – 16:30 | 26:25 | 28:01 | 2.3 | 22.0 |
| Erssjön | 2018-07-24 | 13:14 – 14:20 | 2018-07-25 | 14:05 – 14:59 | 23:45 | 24:36 | NA | 23.5 |
| Erssjön | 2018-08-14 | 12:49 – 14:14 | 2018-08-15 | 14:03 – 16:01 | 25:14 | 25:47 | NA | 18.8 |
| Erssjön | 2018-08-30 | 12:35 – 13:09 | 2018-08-31 | 13:55 – 14:51 | 25:20 | 25:42 | 1.9 | 16.6 |
| Erssjön | 2018-09-19 | 14:32 – 15:07 | 2018-09-20 | 13:55 – 14:51 | 21:59 | 22:07 | 4.9 | 15.5 |
| Erssjön | 2018-10-03 | 14:37 – 15:29 | 2018-10-04 | 12:43 – 13:32 | 22:00 | 22:11 | 1.1 | 9.2 |
| Erssjön | 2018-10-18 | 10:52 – 11:36 | 2018-10-19 | 11:13 – 14:35 | 24:20 | 27:01 | 0.8 | 11.3 |
| Erssjön | 2018-10-31 | 10:46 – 11:29 | 2018-11-01 | 10:39 – 11:32 | 23:52 | 24:03 | 1.0 | 4.8 |
| Erssjön | 2018-11-14 | 11:49 – 12:08 | 2018-11-15 | 10:38 – 11:07 | 22:49 | 22:49 | 2.1 | 7.5 |

**Table S7:** Floating chamber locations for CO_2_ measurements at Erssjön and Erken.

| **lake** | **sampling spot ID** | **water depth [m]** | **Coordinate N** | **Coordinate E** |
| --- | --- | --- | --- | --- |
| Erken | KHM_01 | 0.5 | 59.51903 | 18.28442 |
| Erken | KHM_02 | 1.5 | 59.51924 | 18.28454 |
| Erken | KHM_03 | 2.8 | 59.51931 | 18.28483 |
| Erken | KHM_04 | 4.5 | 59.51968 | 18.28554 |
| Erken | NJÄ_01 | 0.7 | 59.51373 | 18.38318 |
| Erken | NJÄ_02 | 1.3 | 59.51368 | 18.38257 |
| Erken | NJÄ_03 | 2.6 | 59.51359 | 18.38186 |
| Erken | NJÄ_04 | 4.7 | 59.51299 | 18.38249 |
| Erken | SBA_01 | 0.7 | 59.50139 | 18.38596 |
| Erken | SBA_02 | 1.5 | 59.50143 | 18.3859 |
| Erken | SBA_03 | 2.9 | 59.50143 | 18.38586 |
| Erken | SBA_04 | 4.3 | 59.50218 | 18.38516 |
| Erssjön | A_1 | 0.3 | 58.37289 | 12.16528 |
| Erssjön | A_2 | 0.8 | 58.37291 | 12.16509 |
| Erssjön | A_3 | 1.3 | 58.37284 | 12.16496 |
| Erssjön | A_4 | 1.5 | 58.37279 | 12.16415 |
| Erssjön | B_5 | 0.3 | 58.37105 | 12.16319 |
| Erssjön | B_6 | 0.9 | 58.37118 | 12.16298 |
| Erssjön | B_7 | 1.4 | 58.37123 | 12.16281 |
| Erssjön | B_8 | 2.4 | 58.37117 | 12.16259 |
| Erssjön | C_9 | 0.3 | 58.37005 | 12.15811 |
| Erssjön | C_10 | 0.8 | 58.37001 | 12.15818 |
| Erssjön | C_11 | 1.4 | 58.37004 | 12.15845 |
| Erssjön | C_12 | 1.9 | 58.37005 | 12.1588 |
